# Single-Cell RNA Sequencing Reveals Immune Dysregulation and Candidate Drug Targets in Endometriosis

**DOI:** 10.3390/cells15181702

**Published:** 2026-09-19

**Authors:** Marlene Rezk-Füreder, Matin Kazemi, Ayberk Alp Gyunesh, Sharon D. Bryant, Peter Oppelt, Esma Hamzic-Jahic, Diana Reisinger, Celine Kapper, David Demmel, Angelika Lackner, Kevin Lang, Barbara Arbeithuber

**Affiliations:** 1 Experimental Gynaecology and Obstetrics, Department of Gynaecology, Obstetrics and Gynaecological Endocrinology, Johannes Kepler University Linz, 4040 Linz, Austria; marlene.rezk@gmail.com (M.R.-F.); matin.kazemi@jku.at (M.K.);; 2 Inte: Ligand Software-Entwicklungs und Consulting GmbH, 1070 Vienna, Austria; 3 Department of Gynaecology, Obstetrics and Gynaecological Endocrinology, Johannes Kepler University Linz, Kepler University Hospital, 4040 Linz, Austria; 4 Center for Medical Research, Medical Faculty, Johannes Kepler University Linz, 4040 Linz, Austria; 5 Department of Dermatology and Venereology, Medical Faculty, Johannes Kepler University Linz, 4040 Linz, Austria; 6 University Clinic for Cardiac-, Vascular- and Thoracic Surgery, Medical Faculty, Johannes Kepler University Linz, 4040 Linz, Austria

**Keywords:** endometriosis, single-cell RNA sequencing, immune dysregulation, drug repurposing, HSP90AA1, PLCG2

## Abstract

Endometriosis is a chronic inflammatory disorder affecting 5–10% of women of reproductive age and is associated with chronic pain and infertility. While research has mainly focused on local lesions, systemic immune dysfunction is increasingly implicated in disease pathogenesis. This study employs single-cell RNA sequencing (scRNA-seq) of peripheral blood mononuclear cells (PBMCs) from women with endometriosis and unaffected controls, combined with integrative analysis of publicly available datasets of menstrual blood and endometrial tissues, to uncover immune dysregulation and therapeutic targets in endometriosis. Cell-type composition and pathway alterations were assessed. Upregulated genes were evaluated using pharmacophore modeling, virtual screening, molecular docking of approved drugs, and molecular dynamics simulations to explore therapeutic potential. Immune profiling revealed increased B- and T-cell subsets and widespread inflammatory dysregulation. Notably, *HSP90AA1* and *PLCG2* (linked to endometrial cell survival and angiogenesis) were consistently upregulated across datasets, emerging as biologically plausible candidate drug targets for therapeutic intervention. Computational drug repurposing analysis prioritized upadacitinib, sorafenib, alpelisib, and sulindac as hypothetical candidates for further investigation, with molecular docking identifying favorable predicted binding affinity scores, particularly for HSP90AA1. These results reinforce a systemic, immune-mediated view of endometriosis and nominate HSP90AA1 and PLCG2 as novel candidate targets that now require biochemical, chemical, and in vivo validation.

## 1. Introduction

Endometriosis is a complex disorder affecting around 5–10% of women in their reproductive years [1]. This chronic condition is characterized by the growth of endometrial-like tissue outside the uterus, leading to severe pelvic pain, infertility, and a significant reduction in quality of life [2]. The pathogenesis of endometriosis has been a subject of intensive study, yet it remains poorly understood [3,4]. Several theories have been proposed to explain the ectopic presence of endometrial tissue, including retrograde menstruation, coelomic metaplasia, stem cell involvement, and the embryogenetic (Mullerianosis or embryonic rest) theory [5,6,7]. While retrograde menstruation remains the most widely accepted initiating mechanism, it alone cannot fully explain the selective development of lesions in only certain women, despite the high prevalence of menstrual reflux [8,9]. The embryogenetic theory postulates that misplaced primordial endometrial cells of Mullerian origin may already be present at the end of organogenesis and are subsequently activated postnatally [10]; it is supported by the detection of ectopic endometrium in human female fetuses and by transcriptional signatures related to defective organogenesis in endometriotic lesions [7]. Increasing evidence also highlights the importance of additional factors such as immune dysfunction, resistance to apoptosis, hormonal imbalances, and epigenetic alterations, which may promote the survival and implantation of ectopic endometrial cells [8,9]. Aberrant immune responses may fail to clear displaced tissue, while a pro-inflammatory and angiogenic microenvironment may support lesion establishment and persistence [11]. 

One of the primary challenges in endometriosis research has been the focus on the local environment of the lesions [12]. While the study of lesions is undoubtedly important, it does not account for the systemic nature of endometriosis. Initially thought to be a local disorder of the pelvis, endometriosis is increasingly recognized as having widespread systemic effects, particularly on the immune system [13]. However, the molecular basis of these systemic effects has not been extensively investigated. Recent transcriptomic approaches, especially at the single-cell level, offer new opportunities to identify regulatory pathways involved in immune signaling, inflammation, and angiogenesis processes central to endometriosis pathophysiology [8,14]. Understanding differences in gene expression and function across systemic and local compartments may offer new insights into disease mechanisms and therapeutic opportunities.

To address this gap, we conducted single-cell RNA sequencing (scRNA-seq) of peripheral blood mononuclear cells (PBMCs) from endometriosis patients and unaffected controls, incorporating publicly available datasets derived from eutopic endometrium, ectopic lesions, and menstrual blood. This integrative approach enabled the identification of consistently dysregulated genes across multiple tissue types, allowing the prioritization of signals that were reproducible across systemic and local disease compartments. Rather than relying on PBMC findings alone, the analysis focused on shared transcriptional patterns across tissues and immune cell types to strengthen biological consistency. To further explore the potential of these targets, we applied in silico drug repurposing strategies aimed at identifying clinically approved compounds with predicted efficacy against the dysregulated pathways.

## 2. Materials and Methods

### 2.1. Sample Collection and PBMC Isolation

Participants were recruited at the Kepler University Hospital according to Helsinki guidelines. All samples and data were collected in accordance with the guidelines from the Ethics Commission of the Johannes Kepler University Linz (vote number: 1080/2022) and followed the rules of informed consent, anonymization of samples, and avoidance of any ethical sensitivity that may harm an individual. All experimental processes were in accordance with the Declaration of Helsinki. Women with histologically confirmed endometriosis, determined following excision laparoscopic surgery, were enrolled as “endometriosis” subjects. Unaffected subjects were recruited as “controls” only if diagnostic laparoscopy performed for another indication showed no macroscopic endometriotic lesions and if biopsies taken during the same procedure were histologically negative for endometriosis. Because superficial or occult lesions can escape both laparoscopic inspection and biopsy sampling, the presence of clinically silent endometriosis in controls cannot be excluded with certainty.

Whole blood from two control individuals and two endometriosis patients (see Supplementary Table S1 for patient characteristics) was collected in VACUETTE 2 mL serum tubes (Greiner Bio-One GmbH, Kremsmünster, Austria, #454236). PBMCs were isolated by density gradient centrifugation: blood samples were carefully layered over Histopaque (Sigma-Aldrich, St. Louis, MO, USA, #11191-100ML) and centrifuged at 400× *g* for 30 min at room temperature. The mononuclear cell layer was collected, washed twice with PBS by centrifugation at 400× *g* for 8 min at 24 °C, and resuspended. Cells were counted, adjusted to 5 × 10^6^ cells/mL, and cryopreserved in freezing medium (90% FCS, 10% DMSO (Sigma, Aldrich, St. Louis, MO, USA, #D1435-500ML)) at −80 °C for 24 h before transfer to liquid nitrogen. The PBMC dataset was designed as one of multiple datasets, and it was defined a priori that no gene would be reported on the basis of the PBMC data alone. Instead, PBMC-derived signals were retained only when the same direction of dysregulation was observed in independently generated public menstrual blood (*n* = 20) and endometrial (*n* = 14) datasets. This design increases the plausibility of the reported cross-compartment signals but does not provide statistical power at the level of a validation study, and all effect estimates must be regarded as exploratory.

### 2.2. ScRNA-seq Library Construction and Sequencing Data Analysis

PBMCs from two healthy donors and two endometriosis donors were thawed according to the 10× Genomics protocol (CG00039), achieving >80% cell viability, and scRNA-seq libraries for each of the samples were prepared as follows: PBMCs were diluted to a concentration of 700–1200 cells/µL, and ~8300 cells per reaction were loaded onto a Chromium Single Cell Controller (10× Genomics, Pleasanton, CA, USA). scRNA-seq libraries were constructed using the Chromium Next GEM Single Cell 3′ GEM, Library & Gel Bead Kit v3.1 (10× Genomics, Pleasanton, CA, USA, #PN-1000121) following the manufacturer’s instructions and sequenced on an Illumina NextSeq2000 (Illumina, San Diego, CA, USA). To complement these experimental datasets, publicly available scRNA-seq data from menstrual blood samples (GSE203191 [15]; *n* = 20: controls, *n* = 9; endometriosis cases, *n* = 11) and paired eutopic and ectopic endometrium samples (GSE179640 [16]; *n* = 14: controls, *n* = 3; endometriosis cases, *n* = 11; eutopic = 9, ectopic = 11) were integrated. For clarity, “eutopic endometrium” refers to uterine endometrium obtained from women with endometriosis, whereas “ectopic lesions” refers to extrauterine endometriosis-associated tissue. “Control endometrium” refers to uterine endometrium obtained from participants without endometriosis. In the GSE179640 dataset, eutopic endometrium from women with endometriosis was compared with control endometrium, and ectopic lesions from women with endometriosis were also compared with control endometrium. Because a non-diseased ectopic tissue counterpart is not available, the ectopic lesion comparison reflects both disease status and anatomical tissue site. Preprocessed h5 data provided by the authors were used, and further quality control was omitted. The public datasets were not treated as independent validation cohorts; together with the in-house PBMC dataset, they were used for exploratory cross-compartment discovery and prioritization.

Raw sequencing reads from PBMC samples were processed using Cell Ranger (v6.0.0) [17] with the human reference genome Homo sapiens [1000 Genomes] hg38 No Alt Haps (https://use1-prd-seq-hub-appdata.s3.amazonaws.com/TruSeqAmplicon_v3/GRCh38Decoy-fasta-1.0.0/root.tar.gz (accessed on 10 August 2026)). Cells with ≥60% mitochondrial reads, ≥50% ribosomal reads, ≤60 unique genes (features), or read counts ≤150 or ≥30,000 were excluded. Genes expressed in ≤3 cells were excluded from further analysis. Public datasets were analyzed using Scanpy (v1.9.1) [18] in Python. Data normalization was performed to a target sum of 10,000 counts per cell, followed by log-transformation. Each dataset was processed within the same analytical framework, and downstream comparisons focused on concordant differential-expression patterns rather than isolated single-dataset effects. Highly variable genes were identified (minimum mean = 0.0125; maximum mean = 3; minimum dispersion = 0.5), and the data were scaled, capping values at ± 10. Principal component analysis (PCA) was conducted for dimensionality reduction, and a neighborhood graph was computed. Uniform Manifold Approximation and Projection (UMAP) was used for visualizing cell clusters. Symptomatic samples without defined diagnosis were excluded from the menstrual blood dataset prior to downstream analysis. Leiden clustering was applied to identify cellular subpopulations. Dimensionality reduction was recalculated using batch-corrected PCA components, followed by UMAP visualization. Differential gene expression analysis was performed using rank_genes_groups. The top 25 marker genes associated with the specific cluster were used for annotation. Clusters were annotated using the top 25 marker genes and validated against the established literature, with assistance from GPT-4 by OpenAI [19].

Preprocessed matrices provided by the original authors were used for the public datasets, and the cell-level quality filters applied prior to deposition were not modified. All subsequent processing steps were applied identically to all datasets: normalization to 10,000 counts per cell, log transformation, identification of highly variable genes, scaling with values capped at +/−10, principal component analysis, neighborhood graph construction, batch correction, Leiden clustering, and annotation of clusters on the basis of their top 25 marker genes. Cell-type annotations were therefore generated de novo within a single framework and were not adopted from the original publications. Because differential expression was computed within each dataset against the control group of that dataset, differences in upstream filtering affect the sensitivity of an individual dataset but do not enter the comparison between datasets, which uses only the dataset-internal significance calls and their direction.

### 2.3. Identification of Dysregulated Genes Across Immune Cell Types and Tissues

Differential gene expression analysis was conducted using Scanpy’s rank_genes_groups function. Specific cell types, including monocytes, T cells, B cells, and natural killer (NK) cells, were assigned based on characteristic gene expression patterns. Clustered data were visualized in UMAP space, highlighting cell-type abundances and their distributions across conditions (control vs. endometriosis). Cross-tabulated cell counts by condition were generated and exported for comparison. Integration of experimental and public datasets ensured consistency, enabling the identification of transcriptomic patterns reproducible across multiple tissues, thereby reducing reliance on any single dataset. Differential expression was assessed separately for each combination of immune cell type and compartment, comparing endometriosis with control samples. This resulted in 18 cell-type x compartment datasets, corresponding to five immune cell types (T cells, B cells, NK cells, dendritic cells and monocytes) across four compartments (PBMCs, menstrual blood, eutopic endometrium, and ectopic endometrium); monocytes were annotated in PBMCs and menstrual blood only. Within each dataset, genes were considered significant at a Benjamini–Hochberg adjusted *p*-value below 0.05. Results were then merged across datasets, and a gene was retained in the core set only if it (i) reached significance in at least one cell type in each of the four compartments and (ii) showed a consistent direction of regulation across the datasets in which it was significant. Selection was therefore based on reproducibility across biologically independent compartments rather than on the statistical strength of any individual dataset, limiting potential basis due to the small size of the PBMC cohort. This procedure yielded a core set of 118 genes (Supplementary Data S1; 78 upregulated, 40 downregulated), which were significant in a median of 6 of the 18 datasets (range 4–13); 59 of them were significant in at least four of the five immune cell types. All intermediate quantities underlying the selection—the datasets, compartments and cell types in which each gene was significant, the numbers of datasets showing up- and downregulation, the effect sizes per compartment and the adjusted *p*-values—are reported gene by gene in Supplementary Data S2.

To determine which of these genes were not only widely dysregulated but also functionally relevant, a Gene Ontology (GO) analysis was performed using the clusterProfiler R package. This analysis identified significantly affected biological processes, cellular components, and molecular functions. Kyoto Encyclopedia of Genes and Genomes (KEGG) pathway analysis was conducted using g:GOSt from the g:Profiler suite (https://biit.cs.ut.ee/gprofiler/gost, accessed on 1 December 2024). Results were visualized using bar plots, scatter plots, and pathway diagrams generated in R. To further prioritize functional genes, we quantified the frequency of each gene’s involvement in dysregulated GO terms or KEGG pathways. Genes most frequently associated with these functional categories were identified as key targets. This approach resulted in a final list of candidate genes for potential drug repurposing.

### 2.4. Drug Repurposing

ScRNA-seq had identified 118 differentially expressed genes, from which *HSP90AA1* and *PLCG2* were selected for subsequent in silico analysis based on both biological relevance and computational tractability. Biological prioritization was based on the transcriptomic and functional analyses described above, whereas computational tractability was assessed independently according to the availability of suitable experimentally determined protein structures, defined ligand-binding sites, and sufficient known ligand/inhibitor data for pharmacophore modeling and virtual screening. These structural and ligand-data criteria were used solely to determine the feasibility of the computational analysis and were not considered evidence of greater causal relevance to endometriosis. For the in silico studies, HSP90AA1 and PLCG2 3D X-ray diffraction-resolved structures were obtained from the Protein Data Bank (RCSB.org) with PDB IDs 2XJX and 8T7C, respectively [20,21]. The availability of high-resolution crystal structures for these proteins facilitated in-depth computational modeling and enabled structure-based drug discovery through virtual screening, molecular docking and pharmacophore modeling approaches. Using LigandScout version 4.5 Advanced (https://www.inteligand.com/ligandscout/, accessed on 16 December 2024), protein structures were prepared, water molecules were removed, and active sites were defined for in silico experiments. 3D-chemical feature-based pharmacophore models for PLCG2 and HSP90AA1 were developed using LigandScout 4.5 Advanced with default settings using the direct approach [22]. Known inhibitors for both proteins were obtained from publicly available databases such as ChEMBL (https://www.ebi.ac.uk/chembl/, accessed on 16 December 2024) and DrugBank (https://go.drugbank.com, accessed on 16 December 2024) and analyzed to identify key interaction features within their active sites. The models incorporated hydrogen bond donors, hydrogen bond acceptors, hydrophobic regions, and aromatic interactions essential for ligand binding. To enhance predictive accuracy, both shared feature-based models and merged feature pharmacophore models were created for different clusters of inhibitors. These final pharmacophore models were then used as templates for virtual screening against a library of approved drugs.

The virtual compound library included all approved drugs classified under ATC codes G (genito-urinary system and sex hormones), H (systemic hormonal preparations), L (antineoplastic and immunomodulating agents), A (alimentary tract and metabolism), and M (musculoskeletal system). This subset of drugs was chosen for its potential relevance to key pathways involved in endometriosis, such as inflammation, immune dysregulation, and hormonal imbalance. To prepare the drug library for 3D-pharmacophore screening in LigandScout, 3D conformations were generated using iCon (default settings), and the library was calculated using the algorithm idbgen (default setting). Pharmacophore models for PLCG2 and HSP90AA1 were utilized to screen the selected compound library in LigandScout. The screening algorithm ranked compounds based on the LigandScout pharmacophore fit score. Hit drug molecules identified by both target models were prioritized to explore drug molecules with potential dual-inhibitory activity. The virtual screening process generated an initial list of 24 hit compounds.

Prioritized hit compounds from the pharmacophore screening were further analyzed for each target by molecular docking utilizing AutoDock Vina 1.1 [23]. Protein structures were prepared by adding hydrogen atoms and assigning Gasteiger charges, with the docking grid positioned over the active site residues of HSP90AA1 and PLCG2. Each ligand–target pair was subjected to five separate docking runs. The resulting values were treated as predicted binding affinity scores generated by the docking algorithm and were used for relative computational prioritization rather than as experimentally determined binding affinities. LigandScout was used to analyze the predicted docking poses and protein–ligand interaction patterns within the binding sites. Predicted hydrogen-bonding, hydrophobic, and π–π interactions with binding-site residues were considered during compound prioritization. Representative poses of the highest-ranked compounds were visually inspected for their positioning and interactions within the binding sites. The final compounds were prioritized based on their predicted binding affinity scores, pharmacophore fit scores, and predicted interaction profiles. Known inhibitors obtained from ChEMBL and DrugBank were used for pharmacophore model construction and were not used as an independent docking-validation set. No co-crystallized ligand redocking or separate active/inactive docking-control set was performed. Alternative receptor protonation states and ligand tautomeric states were not systematically evaluated. 

### 2.5. Molecular Dynamics Simulations and Trajectory Analysis

Molecular dynamics (MD) simulations were performed for selected docked complexes (holo) and the corresponding apo proteins using GROMACS (version 2024.4) [24]. Protein topology was generated with the CHARMM36 additive force field for proteins [25], and ligand parameters were obtained via the CGenFF workflow [26]. Each system was placed in a triclinic box, solvated with explicit TIP3P water (CHARMM-compatible implementation) [27], neutralized, and adjusted to 0.15 M NaCl, followed by steepest-descent energy minimization until the maximum force fell below 1000 kJ mol^−1^ nm^−1^. Equilibration consisted of a constant-volume (NVT) phase followed by constant-pressure (NPT) dynamics at 310 K and 1 bar using a velocity-rescaling thermostat [27,28] and the Parrinello–Rahman barostat [29] with coupling time constants of 0.1 ps and 2.0 ps, respectively. All bonds involving hydrogen were constrained with LINCS, enabling a 2 fs integration time step. Long-range electrostatics were treated with the particle mesh Ewald (PME) method [30], and van der Waals interactions were switched off between 1.0 and 1.2 nm. For each apo and holo system, three independent production simulations of 100 ns were run using different initial velocity seeds. Trajectories were corrected for periodic boundary conditions and recentered on the protein prior to analysis, and quantitative analyses were performed on the 10–100 ns production window. Global structural stability was characterized by backbone RMSD, and local flexibility by per-residue Cα RMSF. Ligand-binding behavior in holo simulations was evaluated using the protein–ligand center-of-mass (COM) distance and the number of protein–ligand hydrogen bonds over time, complemented by additional contact-based metrics for qualitative inspection of binding and displacement events.

### 2.6. Statistical Analyses 

Phyton (version 3.13.1) was used for the identification of differentially expressed genes (DEGs) and presentation of UMAP plots (as described in more detail in the scRNA-seq library construction and sequencing data analysis). R (version 4.2.0) was used for downstream statistical analyses and graphic representation of the downstream analysis. Barplots, heatmaps, and dendrograms were created with the gplots package [31]. Gene enrichment analysis was carried out with the clusterProfiler package [32] using DEGs that were dysregulated in at least half of the datasets.

## 3. Results

### 3.1. Cell Type Abundance Reveals an Altered Immune Landscape in Endometriosis Patients 

**Figure 1:**
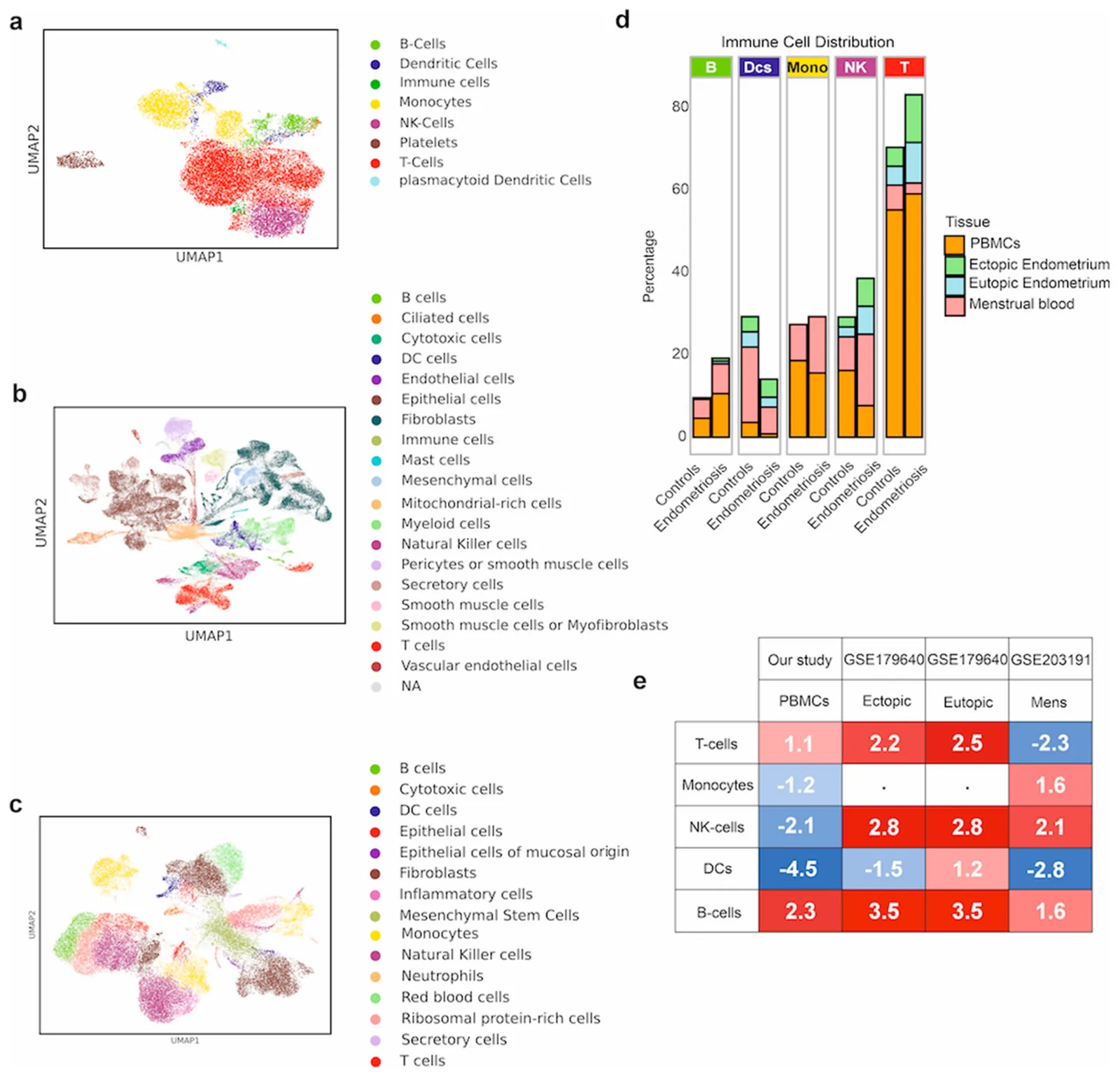
Single-cell transcriptome profiling of control and endometriosis patients. (**a**–**c**) UMAP plots display cell clustering in PBMCs (**a**), eutopic and ectopic endometrium (**b**), and menstrual blood (**c**). Each color represents a distinct cell population, including B cells, dendritic cells (DCs), NK cells, monocytes, and T cells. (**d**) The stacked bar plot shows the relative abundance of immune cell types across different tissue types (PBMCs, eutopic and ectopic endometrium, and menstrual blood) in endometriosis patients and controls. See Supplementary Table S2 for exact numbers of identified immune cells used for downstream analysis, and Supplementary Tables S3–S5 for donor-level information in the PBMC cohort. (**e**) Heatmap comparing the enrichment/depletion of immune cell types in endometriosis across datasets. Red indicates increased abundance, while blue indicates decreased abundance relative to controls.

Cell populations were identified in both control and endometriosis samples, encompassing PBMCs, endometrial tissue, and menstrual blood, consisting of 18, 45, and 26 clusters in the different sample types, respectively (Figures S1–S3). In PBMCs, eight distinct cell types were successfully annotated, including the broadly categorized “immune cells”: UMAP (Figure 1a) revealed distinct clusters corresponding to various immune cell types, including T cells, B cells, monocytes, NK cells, dendritic cells, and platelets. T cells represented the largest cluster (red), followed by monocytes (yellow) and B cells (green). This composition reflects the expected immune profile of PBMCs [33] and provides a baseline for comparing immune cell distribution in other sample types.

In endometrial tissue, 19 different cell types were annotated, including the broadly categorized “immune cells”. One additional cluster remained unclassified. UMAP analysis of eutopic and ectopic endometrial tissue (Figure 1b) identified a range of cell types, including epithelial cells, stromal fibroblasts, mesenchymal stem cells, and inflammatory cells. Notably, monocytes were not evaluated in this dataset. Eutopic samples exhibited a balanced representation of epithelial and stromal cell clusters. In contrast, in ectopic tissue, inflammatory cells and mesenchymal populations, including ribosomal protein-rich cells and vascular endothelial cells, were enriched. Inflammatory clusters were more prominent in ectopic tissue, highlighting the immune dysregulation and stromal remodeling associated with endometriosis pathology. In menstrual blood, 15 different cell types were detected: UMAP visualization (Figure 1c) revealed a diverse cellular composition reflective of its mixed origin from the endometrium and immune system. Epithelial cells and fibroblasts formed prominent clusters, consistent with the shedding of endometrial tissue. Immune cell types, including T cells, B cells, NK cells, and macrophages, were also present, suggesting an active immune environment. Mesenchymal cells and smooth muscle-derived cells were detected as well, likely representing stromal and vascular components of the endometrial tissue.

Comparisons between endometriosis patients and control samples revealed distinct differences in immune cell distribution across all tissue types (Figure 1d,e). Remarkably, B cells were consistently more abundant in endometriosis patients. In both eutopic and ectopic endometrium, B-cell proportions showed a threefold increase (0.7% in endometriosis vs. 0.2% in controls). A similar elevation was observed in menstrual blood (7.2% in endometriosis vs. 4.6% in controls) and was most pronounced in PBMCs (10.6% in endometriosis vs. 4.6% in controls). T cells were also elevated in endometriosis patients, particularly in endometrial tissues and PBMCs. In eutopic endometrium, their proportion nearly doubled (9.9% in endometriosis vs. 4.6% in controls), and a similar increase was seen in ectopic tissue (11.6% in endometriosis vs. 4.6% in controls). A modest increase was noted in PBMCs (59.1% in endometriosis vs. 55.2% in controls). In contrast, menstrual blood showed a reduced T-cell proportion in endometriosis patients (2.6% in endometriosis vs. 6.0% in controls). NK cells showed variable trends: their levels were elevated in both eutopic (6.8% in endometriosis vs. 2.4% in controls) and ectopic endometrium (6.8% in endometriosis vs. 2.4% in controls) and markedly increased in menstrual blood (17.3% in endometriosis vs. 8.2% in controls); However, a significant reduction was observed in PBMCs (7.7% in endometriosis vs. 16.2% in controls). Dendritic cells (DCs) were generally decreased in endometriosis: in eutopic endometrium, their proportion dropped to 2.4% in endometriosis compared to 3.7% in controls; a slight increase was observed in ectopic endometrium (4.4% in endometriosis vs. 3.7% in controls), while notable decreases occurred in menstrual blood (6.5% in endometriosis vs. 18.3% in controls) and PBMCs (0.8% in endometriosis vs. 3.6% in controls). Monocyte data were not available for endometrial tissues. However, in menstrual blood, endometriosis patients showed a marked increase (13.7% in endometriosis vs. 8.8% in controls), while a slight reduction was seen in PBMCs (15.6% in endometriosis vs. 18.6% in controls). 

These findings highlight an altered immune landscape in endometriosis patients compared to controls, with tissue-specific and systemic changes in immune cell distribution. Although the PBMC cohort was limited, the recurrence of related immune shifts across menstrual blood and endometrial datasets supports the broader biological relevance of these patterns.

### 3.2. Transcriptomic Similarities of Immune Cell Types in Endometriosis: Conserved and Tissue-Specific Signatures

**Figure 2:**
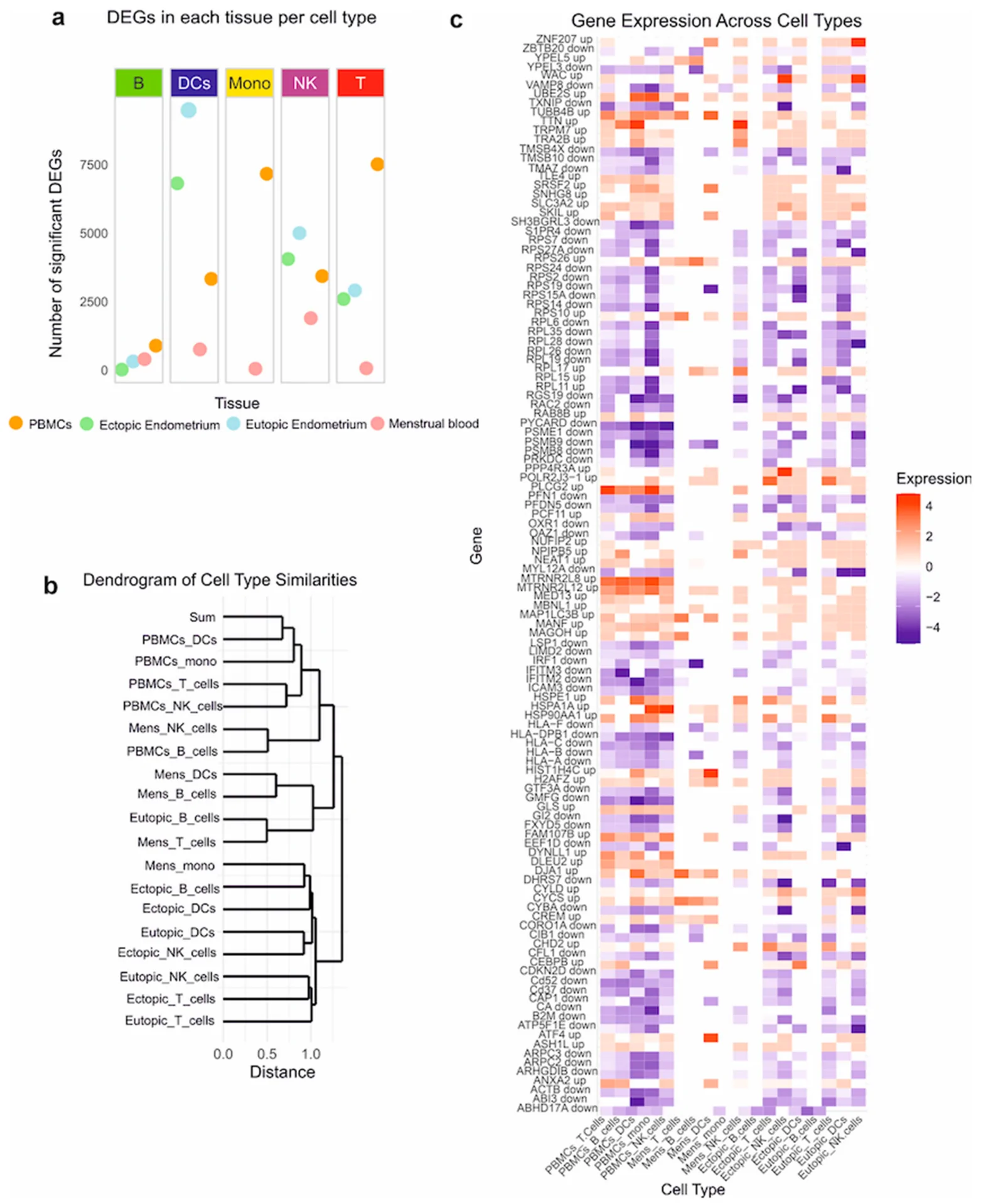
Immune cell transcriptomic alterations in endometriosis patients. (**a**) The dot plot displays the numbers of differentially expressed genes (DEGs) in various immune cell types across different tissues. Each box represents an immune cell type, while the *y*-axis indicates the number of significant DEGs. Dot colors represent the different tissue types (PBMCs, ectopic endometrium, eutopic endometrium, and menstrual blood). (**b**) Hierarchical clustering dendrogram illustrating the transcriptomic similarities between immune cell types across tissues. The *x*-axis shows the distance metric used for clustering, with shorter distances indicating greater similarity. (**c**) Heatmap showing the expression levels of 118 selected genes across different immune cell types and tissues. The color scale represents gene expression levels, with red indicating upregulation and purple indicating downregulation.

The analysis of significant DEGs (Data S1) across various immune cell types and tissues revealed distinct transcriptional patterns in endometriosis (Figure 2a). B cells exhibited the highest number of DEGs in PBMCs (893), followed by menstrual blood (401) and eutopic endometrium (318). In contrast, ectopic endometrium showed a low differential expression in B cells with only 18 DEGs, suggesting limited transcriptional changes in B cells in this tissue compared to systemic (PBMCs) and menstrual compartments. T cells displayed extensive transcriptional changes, with 7515 DEGs in PBMCs—the highest among all tissues analyzed. Both eutopic and ectopic endometrium showed similarly high numbers of DEGs (2911 and 2594, respectively), indicating strong transcriptional responses in endometrial tissues. In menstrual blood, however, only 68 DEGs were detected, reflecting minimal T-cell transcriptional changes in this tissue. NK cells demonstrated the greatest transcriptional activity in eutopic endometrium (5003 DEGs), followed by ectopic endometrium (4054). PBMCs and menstrual blood showed lower DEG counts (3433 and 1896, respectively), suggesting higher transcriptional engagement of NK cells in endometrial tissues. DCs were most transcriptionally active in eutopic endometrium, with 9491 DEGs, followed by ectopic endometrium (6819). Fewer DEGs were observed in PBMCs (3331) and menstrual blood (757) (Figure 2a), indicating that DCs are more active in endometrial environments. Monocytes exhibited 7167 DEGs in PBMCs, reflecting substantial transcriptional changes in systemic circulation. In menstrual blood, only 51 DEGs were observed, indicating limited monocyte activity. Data for monocytes in eutopic and ectopic endometrium were not available. Taken together, these findings show that T cells and monocytes were highly transcriptionally active in PBMCs, pointing to systemic immune activation in endometriosis. In contrast, DCs and NK cells showed the greatest DEG counts in eutopic and ectopic endometrium, underlining their involvement in localized immune responses. Menstrual blood consistently exhibited the fewest DEGs across all cell types, suggesting lower levels of transcriptional change in this tissue.

Analysis of cell type similarities revealed transcriptomic relationships among immune cell populations across tissues in endometriosis (Figure 2b). T cells and B cells from PBMCs clustered together, reflecting their shared lymphoid lineage and systemic distribution. In contrast, immune cells derived from eutopic and ectopic endometrial tissues formed distinct clusters, indicating tissue-specific transcriptomic profiles and potential functional divergence in disease pathology. Notably, eutopic and ectopic immune cells clustered together but remain distinct from circulating immune cells, suggesting a localized immune niche in the endometrial tissues. DCs and NK cells from eutopic and ectopic endometrium also formed distinct clusters, reinforcing the unique molecular signatures of immune cells within the endometrium. Immune cells derived from menstrual blood displayed an intermediate clustering pattern, bridging PBMCs and endometrial tissues. NK cells from menstrual blood clustered closer to PBMC-derived NK cells, while menstrual blood T and B cells group between PBMC and endometrial cell types. These patterns support the idea that menstrual blood, while heterogeneous and transient, retains transcriptomic features of both circulating and endometrial immune cells.

Shared expression trends across immune cell types, including T cells, B cells, NK cells, DCs, and monocytes, further support the hypothesis of systemic immune dysregulation in endometriosis. For example, *MAP1LC3B* was upregulated across most PBMC-derived cell types, suggesting a possible role in autophagy or immune activation. Conversely, genes such as *ARPC2* and *GMFG* were primarily dysregulated in ectopic endometrial cells, indicating their specific relevance to local tissue dysfunction. To identify robust gene signatures, we applied a stringent cross-compartment filter: a gene was retained only if it reached significance in at least one immune cell type in each of the four compartments and if its direction of regulation was consistent across the datasets in which it was significant (Data S2). This filter yielded a core set of 118 genes (78 upregulated, 40 downregulated), significant in a median of six of the 18 cell-type x compartment datasets (range 4–13), with 59 genes significant in at least four of the five immune cell types. Because significance in all four compartments was a mandatory criterion, every gene of the core set is supported by at least three significant observations outside the PBMC compartment (median 4), and the identical set would be obtained if the in-house PBMC data were removed from the selection; the PBMC cohort adds systemic context but does not define the gene set. The core set thus represents systematically affected immune-related transcriptional programs that are reproducible across circulating, menstrual, and endometrial compartments, representing a core set of systematically affected immune-related transcriptional programs in endometriosis. When analyzing expression profiles across tissues and immune cell types for those 118 genes (Figure 2c), we observed both conserved dysregulation patterns and tissue-specific expression signatures. For instance, menstrual blood-derived cells showed unique expression profiles distinct from other tissues, while eutopic and ectopic endometrium-derived cells shared overlapping but distinct gene expression patterns. These findings reflect the diverse roles of local and systemic immune cells in the pathophysiology of endometriosis.

### 3.3. Functional Annotation Supports the Theory of a Systemic Impact of Endometriosis on Immune Cell Activity

**Figure 3:**
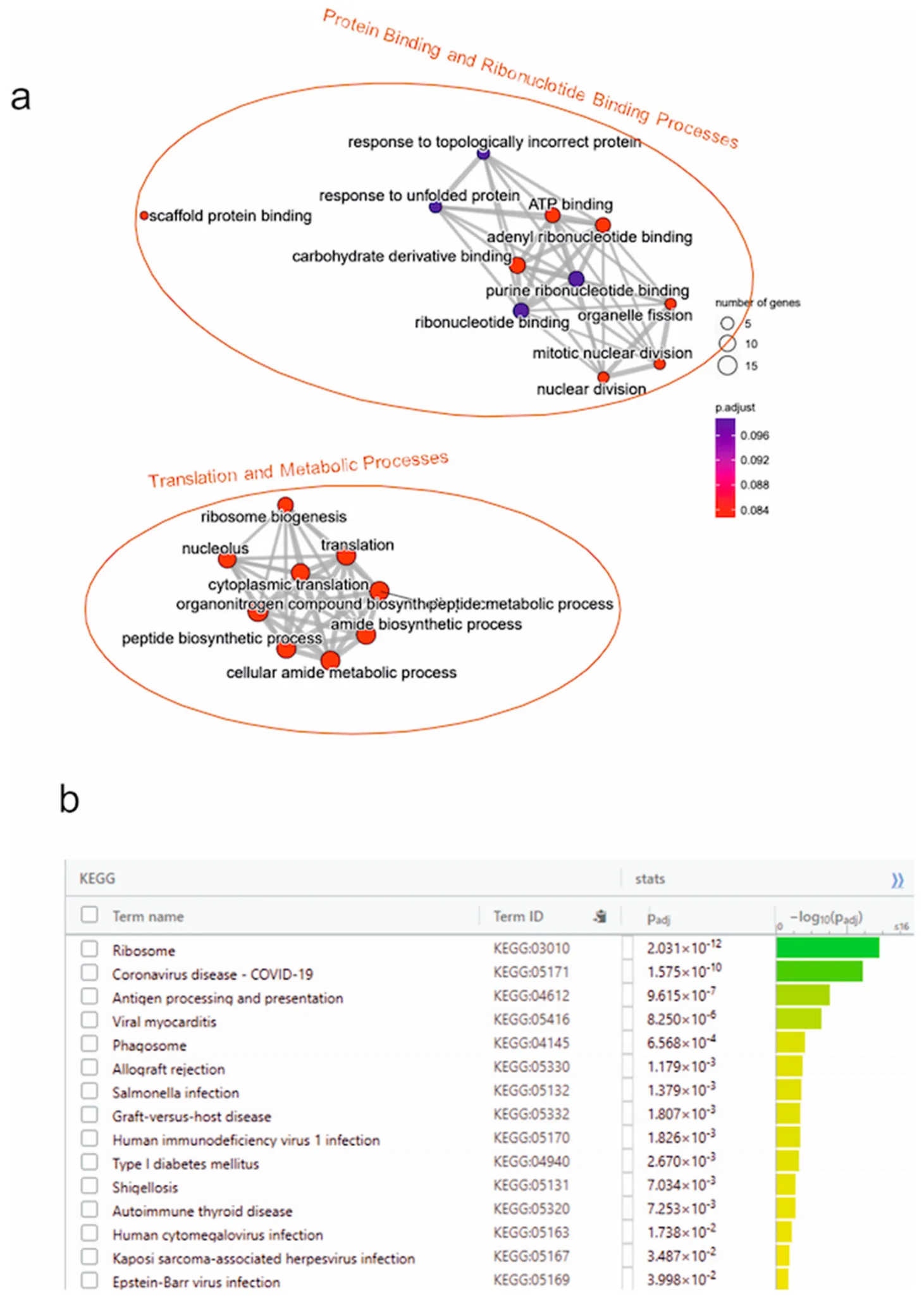
Functional enrichment analysis of 118 target genes. (**a**) Gene Ontology (GO) enrichment analysis on the set of 118 target genes. Results are visualized as a network plot, highlighting significantly enriched biological processes. The identified pathways were grouped into major functional clusters; node size is proportional to the number of associated genes, and node color represents the adjusted *p*-value, with red indicating lower adjusted *p*-values (stronger enrichment) and purple indicating comparatively higher adjusted *p*-values. Grey lines connect GO terms that share overlapping gene sets, indicating functional and gene-set similarity between terms. (**b**) KEGG pathway analysis identified significantly enriched pathways among the 118 target genes. The ranked list displays pathways sorted by statistical significance (*p*-adjusted values), with the top pathways visualized as a bar graph. The green-to-yellow color gradient represents adjusted *p*-value intensity, with green indicating more significant enrichment (lower adjusted *p*-values).

To further elucidate the biological significance of the 118 consistently dysregulated genes, we performed GO and KEGG pathway analyses. These analyses identified key molecular processes, biological functions, and cellular compartments affected in immune cells of endometriosis patients, while also highlighting the most functionally relevant genes. In total, 21 dysregulated GO terms and 16 KEGG pathways were identified, indicating disruptions in protein binding, ribonucleotide binding, translation, and metabolic processes (Figure 3). These pathways are essential for immune system function and cellular metabolism, underscoring the systemic impact of endometriosis on immune cell activity. Among the top 10 functionally significant genes, four were human leukocyte antigen (HLA) genes (*HLA-A, HLA-B, HLA-C*, and *HLA-F*) that were predominantly downregulated. These genes were enriched in pathways such as antigen processing and presentation and autoimmune thyroid disease, suggesting a critical role for impaired antigen presentation and immune suppression in the pathophysiology of endometriosis. Notably, this downregulation may contribute to reduced immune surveillance, impairing the recognition of infected or malignant cells and weakening CD8^+^ T-cell activation. This, in turn, could diminish the immune system’s ability to target abnormal cells. Such a mechanism aligns with the observation that endometriotic cells can persist and proliferate in ectopic locations, potentially evading immune detection—a strategy similarly exploited by tumor cells through HLA suppression [34]. In addition, genes such as *HSPA1A* and *RPS27A* were highly involved across multiple GO terms and KEGG pathways, reflecting their central roles in translation and stress response pathways. *HSPA1A*, which encodes the heat shock protein Hsp70, is particularly important in antigen processing and presentation, contributing to both MHC class I and II pathways. Its upregulation may represent a compensatory mechanism in response to the downregulation of HLA genes, enabling continued antigen presentation and immune activation despite these deficiencies. A comprehensive list of the genes and their associated pathway counts is provided in Supplementary Table S6, with additional details on their properties listed in Data S2. 

### 3.4. Identification of Potential Candidate Therapeutic Compounds Based on Pharmacophore Model Development, Virtual Screening, and Molecular Docking 

From the 10 most predominantly upregulated genes identified before in endometriosis samples (*HSPA1A, HSP90AA1, TTN, HSPE1, UBE2S, CHD2, CYCS, DNAJA1, PLCG2, TRPM7*), we selected *HSP90AA1* and *PLCG2* as promising candidate targets (see Methods for details on the selection process). *HSP90AA1* exhibited a 9-fold upregulation, underscoring its already known involvement in protein stability, cellular stress adaptation, and endometrial cell survival. *PLCG2*, with a 2-fold upregulation, is implicated in angiogenesis, immune regulation, and inflammatory signaling, all of which are critical to endometriosis pathophysiology [35]. Their distinct yet complementary functions in disease mechanisms made them ideal candidates for therapeutic investigation. The remaining top upregulated genes were not prioritized due to either limited suitability for small-molecule targeting, lack of structural data for computational modeling, or broad systemic functions that could lead to undesirable off-target effects. In contrast, HSP90AA1 and PLCG2 had well-defined crystal structures (≤2.6 Å), known ligand-binding sites, and existing pharmacological datasets, enabling their use in structure-based drug discovery [36,37]. To identify potential inhibitors for these targets, eight ligand-based pharmacophore models were constructed (Figure S4). These models were derived from curated libraries of known inhibitors and designed to capture critical molecular interaction features such as hydrogen bond donors, hydrogen bond acceptors, hydrophobic regions, and aromatic interactions. For HSP90AA1 and PLCG2, first, two distinct clusters of inhibitors were identified for each protein and used to generate the ligand-based pharmacophore models. The pharmacophore models for HSP90AA1 were derived from Cluster 7 (LB-HSP90-Cluster7-Mergedfeature and LB-HSP90-Cluster7-Sharedfeature) and Cluster 10 (LB-HSP90-Cluster10-Mergedfeature and LB-HSP90-Cluster10-Sharedfeature). Similarly, the models for PLCG2 were generated based on Cluster 11 (LB-PLCG2-Cluster11-Mergedfeature and LB-PLCG2-Cluster11-Sharedfeature) and Cluster 12 (LB-PLCG2-Cluster12-Mergedfeature and LB-PLCG2-Cluster12-Sharedfeature). These models (Figure S4) highlight critical interaction features such as hydrogen bond donors, hydrogen bond acceptors, hydrophobic regions, and aromatic rings, which are essential for ligand binding. By capturing these key molecular interactions, the pharmacophore models serve as valuable templates for identifying potential inhibitors targeting the active sites of HSP90AA1 and PLCG2. The pharmacophore models were validated to ensure accuracy in predicting potential inhibitors. This validation process demonstrated the robustness of the models in identifying ligands that interact with critical molecular features of the target proteins. Once validated, the pharmacophore models were employed as queries for virtual screening against a library of approved drugs, allowing the identification of compounds with potential inhibitory effects on HSP90AA1 and PLCG2.

The pharmacophore models were then screened against a library of approved drugs sourced from DrugBank, categorized under ATC codes A (alimentary tract and metabolism), G (genito-urinary system and sex hormones), H (systemic hormonal preparations), L (antineoplastic and immunomodulating agents), and M (musculoskeletal system). Additionally, compounds identified from the ChEMBL database, ChEMBL4100 (PLCG2) and ChEMBL2095165 (HSP90), were included in the screening. From this screening, 23 compounds were identified as potential hits, including Niraparib, Upadacitinib, Sorafenib, Sulindac, and Alpelisib. To refine the list of candidates, the compounds were filtered based on their potential relevance to the molecular pathways implicated in endometriosis. Priority was given to compounds targeting inflammation, angiogenesis, and cellular proliferation, which are central to the disease’s pathophysiology [1]. Upadacitinib was selected as a JAK inhibitor due to its ability to modulate immune responses and inflammation [38,39]. Sorafenib, a multi-kinase inhibitor, was prioritized for its anti-angiogenic properties, essential for reducing vascularization in endometriosis lesions [40,41]. Alpelisib, a PI3K inhibitor, was included for its role in cell proliferation and survival pathways [42]. Sulindac, an NSAID (Non-Steroidal Anti-Inflammatory Drug), was selected for its anti-inflammatory effects, which are directly relevant to alleviating endometriosis-associated inflammation [43,44]. After this prioritization process, Upadacitinib, Sorafenib, Alpelisib, and Sulindac emerged as the most promising candidates for further investigation.

**Figure 4:**
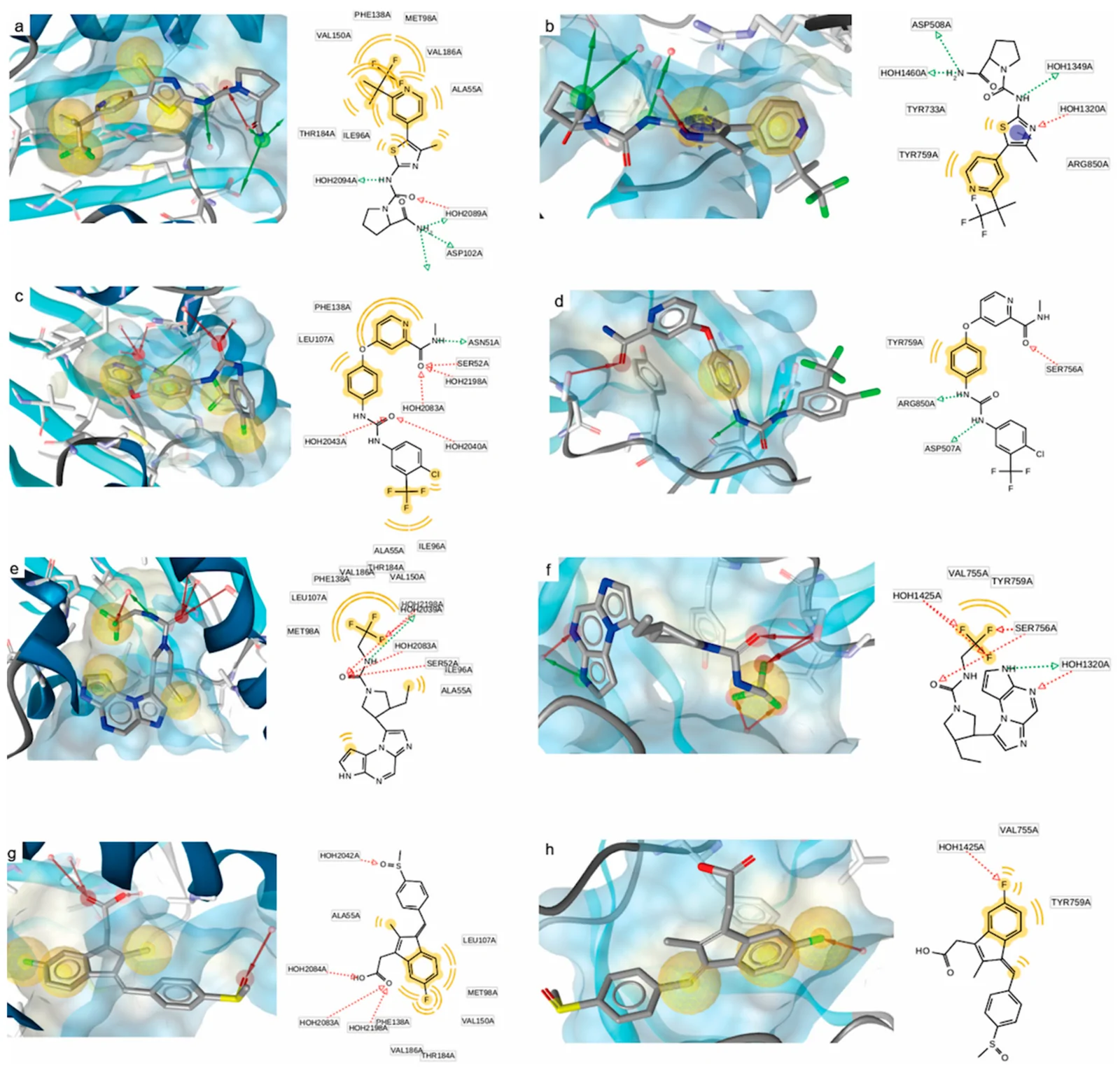
Molecular docking results of selected compounds with HSP90AA1 and PLCG2. (**a**,**b**) Alpelisib: Most favorable predicted binding affinity scores for HSP90AA1 (−34.22 kcal/mol) interacting with Phe138, Thr184, Met98, Leu107, Ala55, and Val186, and predicted binding affinity score for PLCG2 (−13.05 kcal/mol) involving Thr759 and Arg850. (**c**,**d**) Sorafenib: Predicted binding affinity score for HSP90AA1 (−29.35 kcal/mol) with interactions at Val150, Thr184, Ile96, and Ala55, and predicted binding affinity scores for PLCG2 (−16.98 kcal/mol) engaging Thr759 and Arg850. (**e**,**f**) Upadacitinib: Predicted binding affinity scores for HSP90AA1 (−21.08 kcal/mol) with interactions at Phe138, Thr184, Ala55, and Met98, and most favorable predicted binding affinity for PLCG2 (−19.60 kcal/mol) involving Thr759, Tyr733, and Arg850. (**g**,**h**) Sulindac: Predicted binding affinity for HSP90AA1 (−19.29 kcal/mol) interacting with Met98, Ala55, and Thr184, and predicted binding affinity for PLCG2 (−15.28 kcal/mol) showing interactions with Tyr759 and Arg850.

Molecular docking simulations were conducted for the prioritized compounds against HSP90AA1 (PDB ID: 2XJX) and PLCG2 (PDB ID: 8T7C) to evaluate their binding modes and predicted binding affinity scores. The docking results (Figure 4) were interpreted as model-dependent predictions rather than experimentally determined binding affinities. For HSP90AA1, Alpelisib showed the most favorable predicted binding affinity score (−34.22 kcal/mol), with hydrogen-bonding and hydrophobic interactions involving Phe138, Thr184, Met98, Leu107, Ala55, and Val186. Sorafenib (−29.35 kcal/mol) showed interactions with Val150, Thr184, Ile96, and Ala55, while the predicted poses of Upadacitinib (−21.08 kcal/mol) and Sulindac (−19.29 kcal/mol) involved Phe138, Thr184, and Ala55, and Met98, Ala55, and Thr184, respectively. For PLCG2, Upadacitinib showed the most favorable predicted binding affinity score (−19.60 kcal/mol), with interactions involving Thr759, Tyr733, and Arg850. Sorafenib (−16.98 kcal/mol) and Sulindac (−15.28 kcal/mol) showed interactions involving Thr759 and Arg850, whereas Alpelisib (−13.05 kcal/mol) showed the least favorable predicted score among the compounds evaluated for PLCG2. These docking results were used for relative computational prioritization of the selected compounds. 

### 3.5. Molecular Dynamics Simulations to Assess Binding Stability and Interaction Dynamics

**Figure 5:**
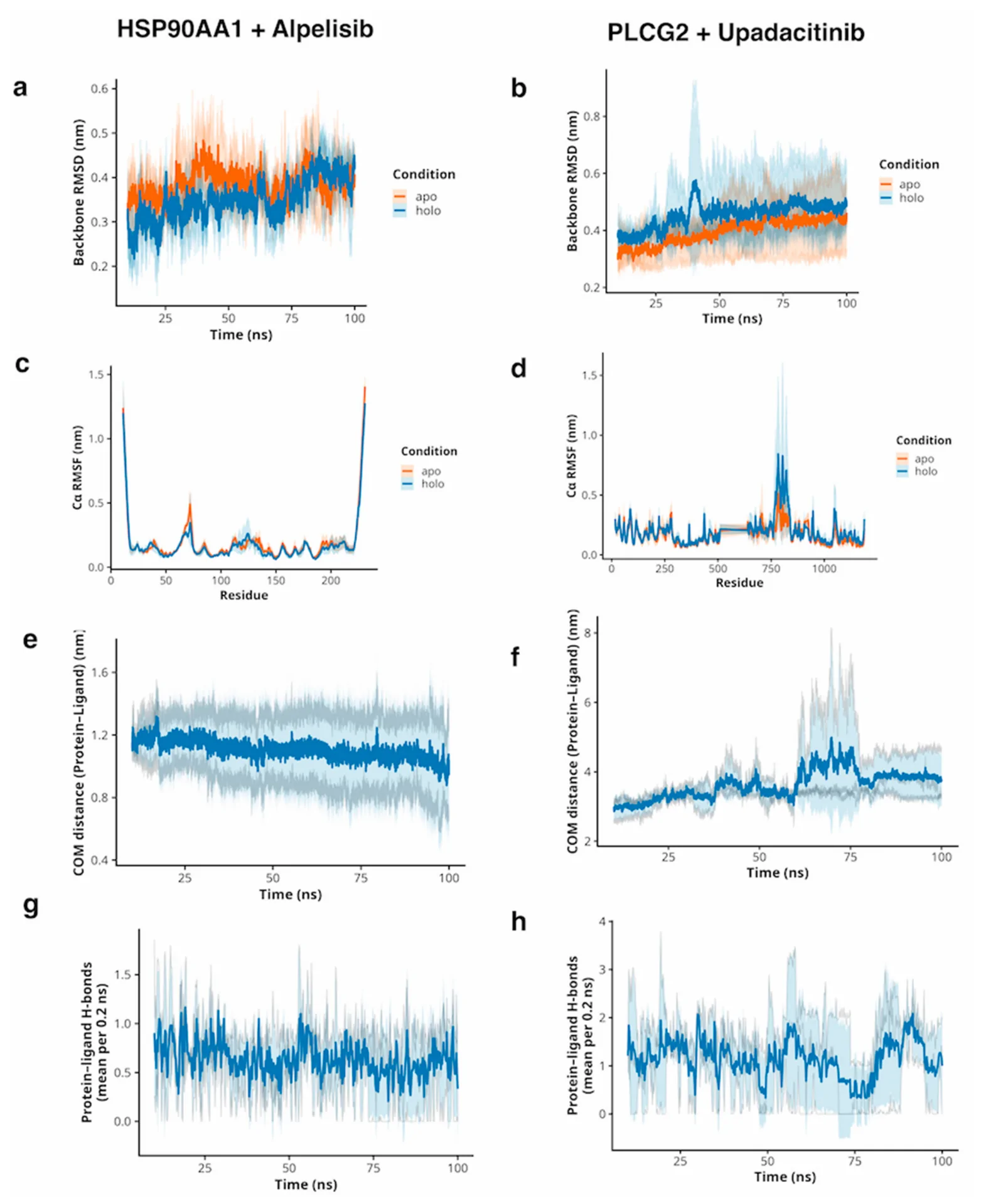
Molecular dynamics analysis of identified drug–target complexes. (**a**,**c**,**e**,**g**) HSP90AA1–Alpelisib and (**b**,**d**,**f**,**h**) PLCG2–Upadacitinib. (**a**,**b**) Backbone RMSD and (**c**,**d**) Cα RMSF comparing apo (orange) and holo (blue) simulations. (**e**–**h**) Ligand stability metrics for holo simulations, showing protein–ligand center-of-mass (COM) distance (**e**,**f**) and hydrogen bond counts (**g**,**h**). Thin lines represent individual replicates (*n* = 3), thick lines indicate the mean, and shaded areas denote the standard deviation. Analyses were performed on the 10–100 ns production window.

While molecular docking identified HSP90AA1 and PLCG2 as candidate targets based on static structures, we next assessed the temporal stability of the corresponding drug–target complexes under dynamic conditions using 100 ns molecular dynamics simulations (Figure 5).

The HSP90AA1–Alpelisib complex exhibited overall high structural stability. Backbone RMSD values for both apo and holo trajectories remained within ~0.3–0.4 nm after equilibration, with only small differences between the two states (Figure 5a). Local flexibility profiles (Cα RMSF) showed low fluctuations across most residues and no pronounced peaks at the binding site (Figure 5c). Consistently, the protein–ligand center-of-mass (COM) distance stayed narrowly distributed around ~1.1–1.3 nm over the full 100 ns window (Figure 5e), and the number of protein–ligand hydrogen bonds fluctuated around roughly one contact on average without extended periods of consistently zero hydrogen bonds (Figure 5g). These observations indicate a compact and persistent binding mode of Alpelisib within the HSP90AA1 pocket.

In contrast, the PLCG2–Upadacitinib complex displayed more dynamic behavior driven by increased protein flexibility. After an initially stable phase, the holo backbone RMSD showed a marked transient increase between ~40 and 60 ns that was not present in the apo simulations (Figure 5b). This coincided with a pronounced RMSF peak in a flexible region around residues ~750–800 (Figure 5d). During this rearrangement, the protein–ligand COM distance increased from ~3.0–3.5 nm to >4 nm and the number of contacts and hydrogen bonds decreased, indicating a temporary displacement of the ligand from the binding pocket (Figure 5f–h). After ~20 ns, the COM distance decreased again and intermolecular contacts were re-established, consistent with ligand re-association (rebinding) in at least part of the trajectories. Together, these data suggest that Upadacitinib forms specific interactions with PLCG2, but that its binding stability is strongly influenced by protein domain flexibility, resulting in transient ligand displacement rather than a permanently locked conformation.

## 4. Discussion

Endometriosis is a chronic, multifactorial condition characterized by persistent inflammation, abnormal angiogenesis, and dysregulated cell proliferation [45]. Identifying molecular targets is essential for developing more effective therapies. This study applies a multi-compartment single-cell strategy to examine systemic and local immune alterations in endometriosis by integrating PBMC, menstrual blood, and endometrial datasets. Integrating data from PBMCs, menstrual blood, and eutopic/ectopic endometrium provides a broader view of immune dysregulation than any single tissue alone. The identification of consistently dysregulated genes across these tissues strengthens the robustness of our findings by reducing false positives and emphasizing shared disease mechanisms. We identified alterations in immune cell-type abundance and transcriptional profiles across several disease compartments. A consistent increase in B cells across all tissues suggests a role in adaptive immune dysregulation. This is supported by prior reports of increased B-cell activity and elevated B-cell activating factor (BAFF) in endometriosis [46]. Our data reinforce B-cell involvement in chronic inflammation and suggest that targeting B-cell responses could be therapeutically beneficial. Elevated T-cell activity in both endometrial tissues and PBMCs indicates systemic immune involvement. This aligns with previous studies highlighting an imbalance between Th1/Th17 and regulatory T cells (Tregs), contributing to exaggerated inflammation and reduced immune tolerance [47]. These findings support the view of endometriosis as a systemic inflammatory disease. The depletion of NK cells in PBMCs and their increased presence in menstrual blood and endometrial tissues suggest altered migration and dysfunction. Prior research showed impaired NK cytotoxicity in endometriosis, allowing ectopic cells to evade immune clearance [48]. Our results support this mechanism and suggest that NK-cell recruitment may occur but with limited functional activity. Clustering analysis revealed systemic and tissue-specific immune signatures. PBMC-derived immune cells (DCs, monocytes, NK cells, T cells, and B cells) clustered together, reflecting their shared origin. In contrast, eutopic and ectopic tissue-derived immune cells formed distinct clusters, consistent with their functional adaptation in local inflammatory environments [1,13]. B cells from eutopic and ectopic tissues clustered together, highlighting a localized immune niche consistent with reports of chronic inflammation and antigen presentation in endometrial lesions [47]. DCs and NK cells from endometrial tissues showed distinct profiles from their PBMC counterparts, reinforcing evidence of altered antigen presentation and impaired immune surveillance [48]. Interestingly, menstrual blood-derived immune cells exhibited an intermediate profile, bridging systemic and local compartments. These findings, consistent with findings by others [15], validate the utility of menstrual blood as a diagnostic and mechanistic sample source in endometriosis research. We identified 118 consistently dysregulated genes across tissues and immune cell types. These genes likely play central roles in immune dysfunction and disease progression. Notably, *HLA-A*, *HLA-B*, *HLA-C*, and *HLA-F* were downregulated and enriched in antigen presentation and autoimmune-related pathways, supporting prior findings linking HLA dysregulation to impaired immune surveillance and increased autoimmune disease risk [49], as seen in the higher prevalence of autoimmune comorbidities in endometriosis [50,51]. Epidemiological studies have also reported associations between endometriosis and certain autoimmune comorbidities; however, the present transcriptomic data do not establish an autoimmune mechanism or a causal relationship between HLA dysregulation and these comorbidities. 

In particular, genes such as *HSP90AA1* and *PLCG2*, which are involved in stress adaptation, immune signaling, and inflammatory pathways, have attracted attention as potential mediators of ectopic cell survival and lesion progression [36,37,52,53]. The availability of structural data enabled pharmacophore modeling, molecular docking, and virtual screening to prioritize drug repurposing candidates for further investigation. In contrast, many other dysregulated genes lacked sufficient structural data for in silico exploration. Given the limitations of current treatments (partial efficacy, adverse effects, and limited systemic impact) our computational approach aimed to identify drug candidates targeting key pathways in endometriosis [54,55]. We focused on HSP90AA1 and PLCG2 due to their relevance and tractability. Using pharmacophore models built with LigandScout, we screened a library of approved drugs and identified 23 hits, eventually prioritizing four: Upadacitinib, Sorafenib, Alpelisib, and Sulindac. These compounds address key mechanisms of endometriosis. Upadacitinib, a JAK inhibitor, reduces inflammatory cytokine signaling and is effective in autoimmune conditions like rheumatoid arthritis [56]. Sorafenib, a multi-kinase inhibitor, blocks angiogenic pathways (VEGFR, PDGFR) critical for ectopic lesion vascularization [40,41]. Alpelisib targets the PI3K/AKT pathway, implicated in cellular proliferation in endometriosis [57,58]. Sulindac, a COX-inhibitor NSAID, reduces inflammatory mediators and has shown efficacy in preclinical endometriosis models [59,60]. Molecular docking confirmed strong binding affinities, particularly for HSP90AA1: Alpelisib (−34.22 kcal/mol), Sorafenib (−29.35), Upadacitinib (−21.08), and Sulindac (−19.29). These compounds interacted with key residues in the ATP-binding pocket of HSP90AA1, such as Leu107, Phe138, Thr184, and Val186 [61]. For PLCG2, binding affinities were lower (consistent with its moderate upregulation), but Upadacitinib still showed strong interaction (−19.60 kcal/mol), primarily at Thr759, a residue essential for enzymatic activity [21]. Although HSP90AA1 appears to be the more dominant target, PLCG2 inhibition may offer complementary benefits by modulating inflammatory and angiogenic pathways. Overall, these compounds act on processes that are relevant to endometriosis pathology—inflammation, immune dysregulation, angiogenesis, and abnormal proliferation—but the evidence presented here places them only in the first of four categories of target-specific evidence, namely in the category of compounds that dock computationally, but not in the categories of compounds with demonstrated biochemical target engagement, compounds with confirmed cellular pathway modulation, or compounds that improve disease phenotypes in vivo. None of the four compounds has been experimentally shown to act through HSP90AA1 or PLCG2; all are approved for other indications with substantial off-target and safety profiles (for example, immunosuppression and thromboembolic risk for upadacitinib, hypertension and hand–foot syndrome for sorafenib, hyperglycaemia for alpelisib), and it is unknown whether concentrations sufficient for target engagement in endometriotic tissue are achievable at approved doses. However, any clinical use would require dedicated preclinical and clinical evaluation.

To complement docking, we performed 100 ns molecular dynamics simulations for the top-scoring complexes HSP90AA1–Alpelisib and PLCG2–Upadacitinib. The HSP90AA1–Alpelisib complex displayed stable backbone RMSD, low local flexibility at the binding site, a narrow distribution of protein–ligand center-of-mass (COM) distances, and persistent hydrogen-bonding contacts, indicating a compact and long-lived binding mode. In contrast, the PLCG2–Upadacitinib complex exhibited a transient increase in RMSD and RMSF in a flexible domain region, accompanied by a temporary increase in COM distance and loss of contacts, followed by ligand re-association. These findings support Alpelisib as a structurally stable binder of HSP90AA1 within our simulation setup, whereas Upadacitinib interacts specifically with PLCG2 but its binding stability is strongly modulated by protein domain flexibility rather than a permanently locked conformation. However, MD-derived binding stability does not directly translate into functional inhibition, and experimental validation will be required to confirm target engagement and downstream pathway modulation.

Our observations may also be considered in the context of embryogenetic theories of endometriosis. If Müllerian-derived cell remnants displaced during fetal organogenesis contribute to lesion initiation, the immune alterations observed here may represent a permissive rather than initiating context [6,7]. Reduced antigen presentation-associated HLA expression, altered NK-cell distribution, and expanded B-cell compartments may be consistent with impaired immune surveillance and could contribute to lesion persistence and a pro-inflammatory, angiogenesis-supportive microenvironment [62]. HSP90AA1 may support cellular stress responses and survival, whereas PLCG2 is involved in immune-receptor signaling and has been linked to angiogenic pathways in other contexts [63,64]. However, our cross-sectional postmenarcheal data cannot distinguish among embryogenetic, retrograde menstruation, metaplasia, and stem cell models.

Study limitations and future directions: While our computational findings are promising, several limitations must be acknowledged. The experimental PBMC cohort comprises two cases and two controls. Integration with public datasets provides biological context and reduces reliance on a single small cohort, but it does not convert a four-donor discovery cohort into an independently powered validation study; consequently, all reported differences are exploratory. Another limitation is the absence of experimental validation of the identified candidate targets at the RNA and protein level. Accordingly, the main strength of the study lies in the convergence of immune and transcriptomic signatures across systemic and local tissue compartments. Moreover, measured mRNA expression might be influenced by the menstrual cycle in individual sample types. For this reason, we used menstrual blood as an independent validation sample type specifically to help exclude cycle-related PBMC effects and to strengthen the biological signal beyond peripheral blood alone. However, because the endometrial dataset contains no annotated monocyte or classical myeloid compartment, monocytes could not be included in the endometrial comparisons, and all monocyte statements in this study are restricted to menstrual blood and PBMCs. This gap is a property of the available annotation rather than a biological finding, and it limits the cross-compartment interpretation for this cell type. Another limitation is the absence of experimental validation of identified targets at the RNA or protein level. Future studies should include techniques such as qPCR, Western blotting, and functional assays in in vitro and in vivo models to confirm the biological relevance of those findings. Transcriptomic analysis, while powerful, does not account for post-translational modifications or actual protein activity. Complementary proteomic studies could help refine the identification of actionable candidate targets. Moreover, docking simulations rely on static protein structures, which may not fully capture dynamic ligand interactions. Experimental validation in vitro and in vivo is necessary to confirm therapeutic potential and safety. Future studies should also evaluate synergistic drug effects: investigating combination therapies targeting both HSP90AA1 and PLCG2 may further enhance therapeutic potential. 

## 5. Conclusions

This study provides an exploratory, hypothesis-generating framework for the prioritization of molecular candidate targets in endometriosis by combining multi-tissue single-cell transcriptomics with structure-based in silico screening. Its principal contribution is prioritization: from 118 concordantly dysregulated genes, two were selected that were repeatedly dysregulated across tissues and cell types and were computationally tractable. The identification of immune-related dysregulation and candidate therapeutic targets indicates that immune-related transcriptional alterations are observed across the analyzed circulating, menstrual, eutopic, and ectopic compartments. These findings are compatible with cross-compartment immune dysregulation in endometriosis. By integrating pharmacophore modeling, virtual screening, and molecular docking, we identified Upadacitinib, Sorafenib, Alpelisib, and Sulindac as promising therapeutic candidates, based on their high binding affinities, mechanistic relevance, and ability to target key pathways involved in disease progression. Our findings support HSP90AA1 as the primary candidate therapeutic target, with PLCG2 modulation serving as a secondary strategy. This study provides a robust framework for drug repurposing, offering a rational approach to developing targeted therapies for endometriosis. Future studies should focus on experimental validation of these targets and exploring their therapeutic potential in preclinical and clinical settings to translate these computational insights into clinical applications.

## Data Availability

ScRNA–seq data that support the findings of this study have been deposited in the Sequencing Read Archive under the accession PRJNA1344611. All other data supporting the findings of this study are available from the corresponding author on reasonable request.

## Supplementary Materials

The following supporting information can be downloaded at https://www.mdpi.com/article/10.3390/cells15181702/s1, Figure S1: Cell clustering analysis with UMAP plots; Figure S2: UMAP plots for endometriosis patients and controls; Figure S3: UMAP plots on donor level in PBMCs; Figure S4: Ligand-based pharmacophore models for HSP90AA1 and PLCG2; Table S1: Patient information; Table S2: Differentially expressed genes; Table S3: Technical and quality-control metrics per PBMC donor; Table S4: Number of cells per cell type, donor and condition (PBMCs); Table S5: Donor-level cell-type proportions (PBMCs); Table S6: GO and KEGG analyses; Data S1: Differential gene expression; Data S2: Detailed properties of differentially expressed genes.
