# Supplementary material for: Single-Cell RNA Sequencing Reveals Immune Dysregulation and Candidate Drug Targets in Endometriosis
Source: Cells. 2026 Sep 19;15(18):1702. doi: 10.3390/cells15181702 (PMC13605760; doi:10.3390/cells15181702)
Supplement: Supplementary file 1 [file cells-15-01702-s001.zip › 0926_Supplements_EndoSc_revised-1.pdf]

Supplementary Information for

Single-cell RNA sequencing reveals immune dysregulation and candidate drug targets in endometriosis

Supplementary figures **2**
Figure S1. Cell clustering analysis with UMAP plots 2
Figure S2. UMAP plots for endometriosis patients and controls 3
Figure S3. UMAP plots on donor level in PBMCs 4
Figure S4. Ligand-based pharmacophore models for HSP90AA1 and PLCG2 5

Supplementary tables **6**
Table S1. Patient information 6
Table S2. Differentially expressed genes 7
Table S3. Technical and quality-control metrics per PBMC donor 8
Table S4. Number of cells per cell type, donor and condition (PBMCs) 11
Table S5. Donor-level cell-type proportions (PBMCs). 12
Table S6. GO and KEGG analyses 12

Supplementary figures

Figure S1. Cell clustering analysis with UMAP plots.
(a) PBMCs, (b) eutopic and ectopic endometrium, and (c) menstrual blood. Each color represents a distinct cluster.

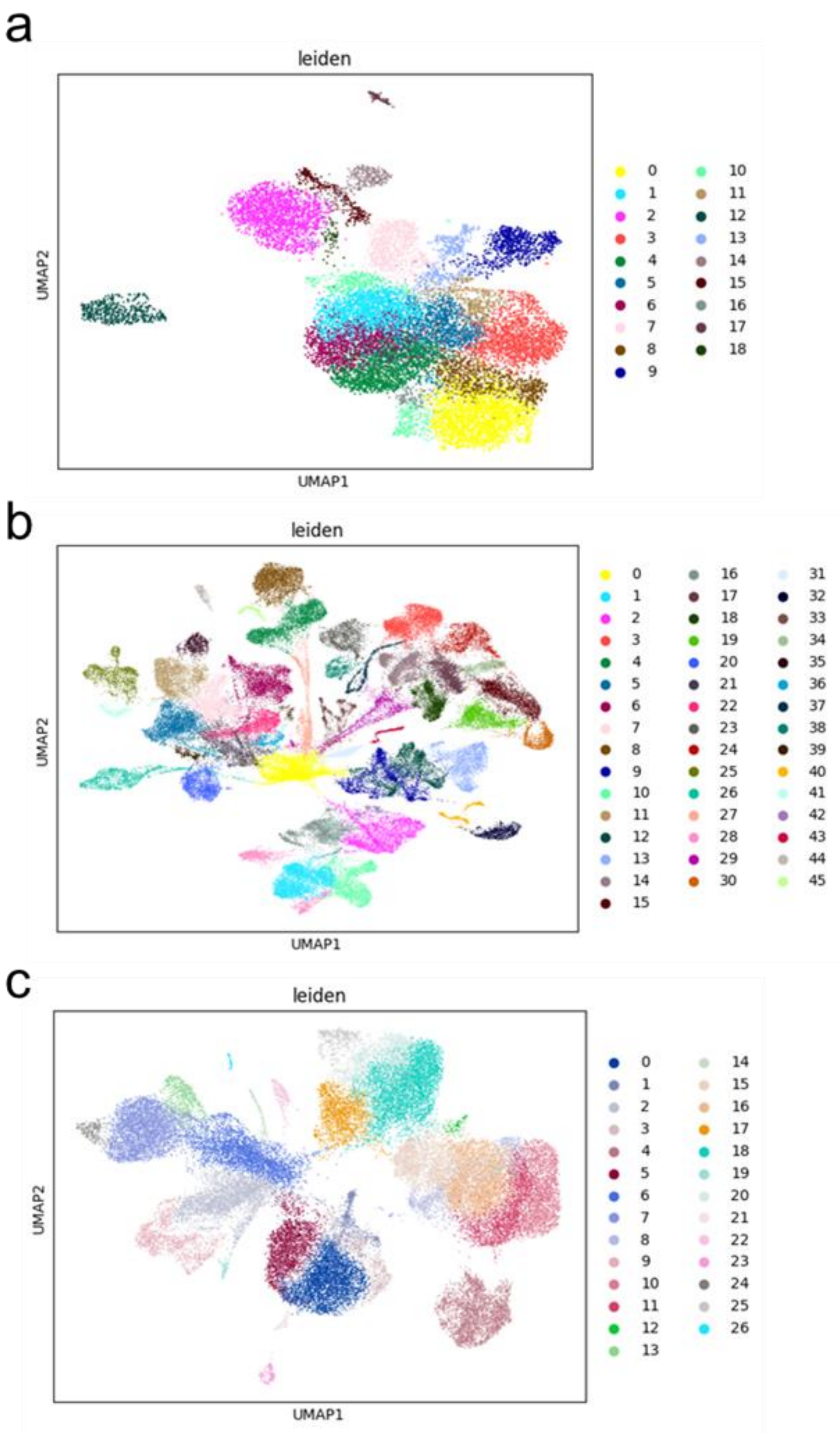

Figure S2. UMAP plots for endometriosis patients and controls.

UMAP plots showing cell clustering of (**a**) PBMCs from endometriosis patients (orange) and control patients (blue); (**b**) Eutopic (green) and ectopic (orange) endometrium from endometriosis patients and endometrium from control patients (blue); and (**c**) and menstrual blood from controls (blue) and diagnosed (orange) and symptomatic (green) endometriosis patients.

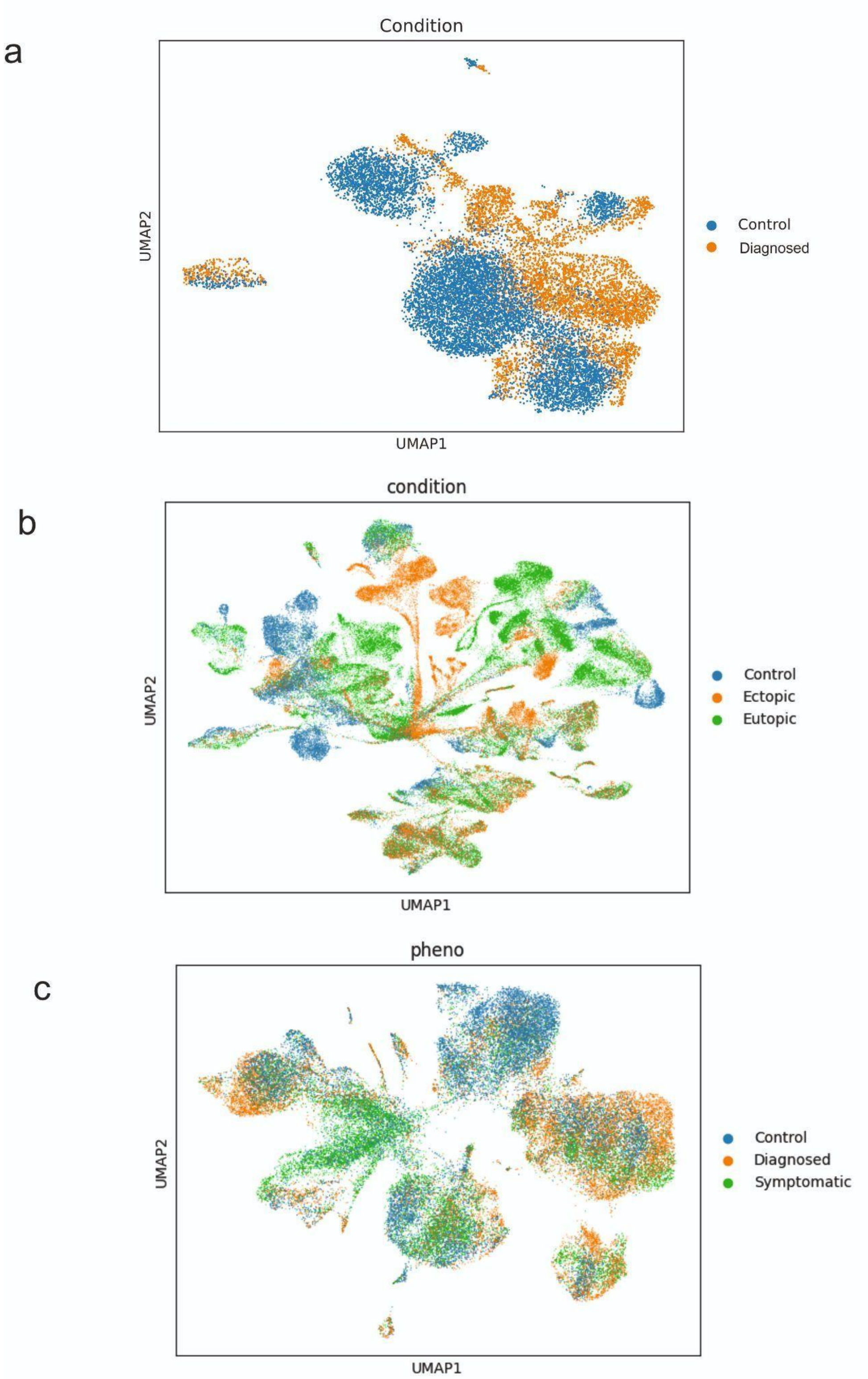

Figure S3. UMAP Plots on donor level in PBMCs.
UMAP plot showing cells of samples with donor ID 24D (endometriosis), 31D (endometriosis), 36C (control) and 43C (control).

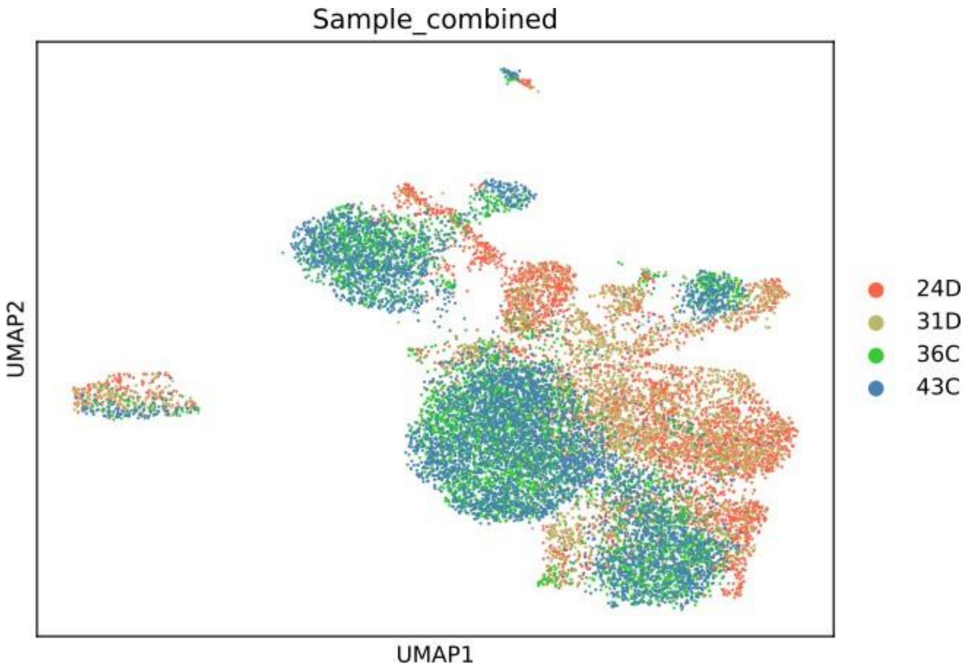

Figure S4. ULigand-based pharmacophore models for HSP90AA1 and PLCG2.
(**a**) Cluster7- Mergedfeature model, (**b**) Cluster7-Sharedfeature, (**c**) Cluster10-Mergedfeature model, and (**d**) Cluster10-Sharedfeature model represent 3D-pharmacophore models for HSP90AA1. (**e**) Cluster11-Mergedfeature model, (**f**) Cluster11-Sharedfeature model, (**g**) Cluster12-Mergedfeature model, and (**h**) Cluster12-Sharedfeature model illustrate pharmacophore models for PLCG2. Pharmacophoric features are highlighted: hydrogen bond donors (green), hydrogen bond acceptors (red), hydrophobics (yellow), aromatic features (blue doughnut), and positive ionizable (blue star). These models were constructed based on known inhibitors of HSP90AA1 and PLCG2, capturing key interaction features and 3D-geometries essential for ligand binding and serving as queries in virtual screening campaigns to identifying potential inhibitors targeting these proteins.

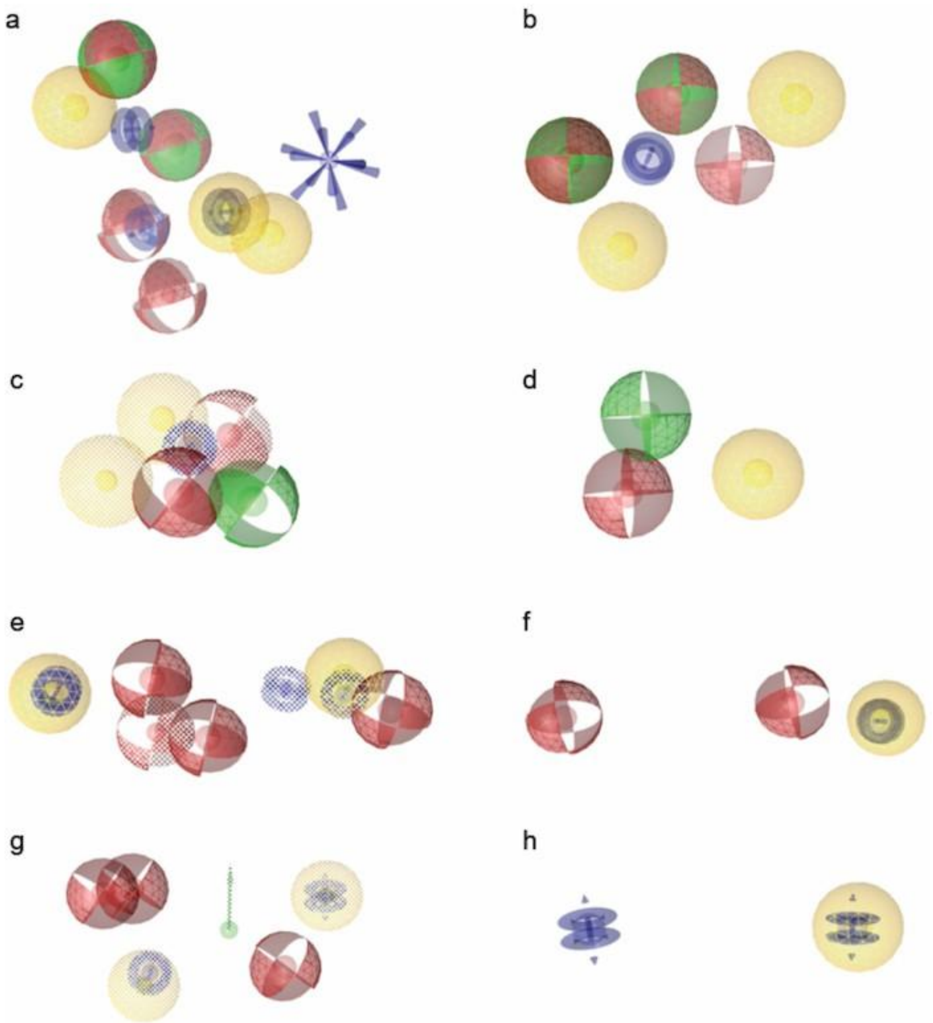

Supplementary tables

Table S1. Patient information.
Characteristics of the patients included for PBMC scRNA-seq.

Tissue type	Group	Included patients (n)	Age [years]: average (standard deviation)	Additional information
PBMC	Endometriosis	2	28.5 (3.5)	1x peritoneal endometriosis; 1x ovarian endometriosis; no hormone use
PBMC	Control	2	33.5 (14.8)	1x ovarian cyst; 1x secondary dysmenorrhea
Menstrual blood (GSE203191)	Endometriosis	11	35.2 (4.4)	not breastfeeding; no hormone use (except for one donor); collection: day 1 or 2, 4-8h [12]
Menstrual blood (GSE203191)	Control	9	33.4 (5.4)	self-reported no gynecologic history suggestive of endometriosis, absence of PMOS and pelvic inflammatory disease; no hormone use; collection: day 1 or 2, 4-8h [12]
Eutopic endometrium (GSE179640)	Endometriosis	9	35.8 (5.8)	oral contraceptive: 8/9; ASRM Stage II-IV [13]
Ectopic endometrium (GSE179640)	Endometriosis	11	35.4 (5.3)	peritoneal (n=8), peritoneal adjacent (n=6), ovary (n=4), multiple localizations were analyzed in 7 of the donors; oral contraceptive: 10/11; ASRM Stage II-IV [13]
Eutopic endometrium (GSE179640)	Control	3	33.3 (10.3)	oral contraceptive: 2/3; [13]

Table S2. Differentially expressed genes.

The number of identified differentially expressed genes (DEGs) found in various immune cell types and tissues when comparing controls to endometriosis samples.

Cell_type	Tissue	Number of DEGs	Control cells	Diseased cells
B-cells	Eutopic Endometrium	318	42	293
B-cells	Ectopic Endometrium	18	42	138
B-cells	Menstrual blood	401	623	870
B-cells	PBMCs	893	443	698
T-cells	Eutopic Endometrium	2911	795	4107
T-cells	Ectopic Endometrium	2594	795	2204
T-cells	Menstrual blood	68	822	313
T-cells	PBMCs	7515	5296	3897
NK-cells	Eutopic Endometrium	5003	413	2824
NK-cells	Ectopic Endometrium	4054	413	1283
NK-cells	Menstrual blood	1896	1121	2077
NK-cells	PBMCs	3433	1555	509
DCs	Eutopic Endometrium	9491	649	980
DCs	Ectopic Endometrium	6819	649	829
DCs	Menstrual blood	757	2504	784
DCs	PBMCs	3331	344	50
Monocytes	Eutopic Endometrium	-	-	-
Monocytes	Ectopic Endometrium	-	-	-
Monocytes	Menstrual blood	51	1201	1653
Monocytes	PBMCs	7167	1788	1028

Table S3. Technical and quality-control metrics per PBMC donor.
Sequencing, alignment and quality-control metrics for each of the four participants included in the PBMC scRNA-seq analysis. All libraries were prepared and sequenced together and processed with a single alignment pipeline version and reference genome.

**Donor ID**	**24D**	**31D**	**36C**	**43C**
Group	Endometriosis (peritoneal)	Endometriosis (ovarian)	Control	Control
Age	26	31	44	23
Cells before QC	3308	2189	4594	4892
Cells after QC	3061	1869	4472	4705
Cells removed (%)	7.5	14.6	2.7	3.8
Doublets removed	0	5	6	52
Median UMI per cell	4107	3824	1325	3385
Median genes per cell	1284	1351	578	1145
Median mitochondrial fraction (%)	8.4	7.09	8.43	9.24
Upper gene threshold (98th pct)	2626	2571	1414	2505
Flowcell	AAFC5FGM5	AAFC5FGM5	AAFC5FGM5	AAFC5FGM5
Sequencing run	240207_VH01205_5	240207_VH01205_5	240207_VH01205_5	240207_VH01205_5
Instrument	VH01205	VH01205	VH01205	VH01205
Alignment pipeline	DRAGEN scRNA v07.021.602.3.8.4	DRAGEN scRNA v07.021.602.3.8.4	DRAGEN scRNA v07.021.602.3.8.4	DRAGEN scRNA v07.021.602.3.8.4
Reference genome	hg38 no-alt with decoy	hg38 no-alt with decoy	hg38 no-alt with decoy	hg38 no-alt with decoy

Table S4. Number of cells per cell type, donor and condition (PBMCs).

Cell type	24D (Endo)	31D (Endo)	36C (Control)	43C (Control)	Endometriosis total	Control total	All donors
B cells	231	272	215	224	503	439	942
Dendritic cells	91	60	131	178	151	309	460
Unassigned immune cells	19	78	4	5	97	9	106
Monocytes	813	219	863	927	1032	1790	2822
NK cells	336	177	762	794	513	1556	2069
Platelets	145	165	59	98	310	157	467
T cells	2368	1584	2580	2709	3952	5289	9241
Plasmacytoid dendritic cells	28	3	16	25	31	41	72
Total cells in the annotated object	4031	2558	4630	4960	6589	9590	16179

Table S5. Donor-level cell- type proportions (PBMCs).

Cell type	24D (Endo) [%]	31D (Endo) [%]	36C (Control) [%]	43C (Control) [%]
NK cells	8.34	6.92	16.46	16.01
T cells	58.74	61.92	55.72	54.62
Monocytes	20.17	8.56	18.64	18.69
B cells	5.73	10.63	4.64	4.52
Dendritic cells	2.26	2.35	2.83	3.59
Plasmacytoid dendritic cells	0.69	0.12	0.35	0.5
Platelets	3.6	6.45	1.27	1.98
Unassigned immune cells	0.47	3.05	0.09	0.1

Table S6. GO and KEGG analyses.

Key genes identified through GO (Gene Ontology) and KEGG (Kyoto Encyclopedia of Genes and Genomes) analyses that show significant up- or down-regulation in endometriosis. Each entry lists the number of enriched biological processes and pathways associated with each gene. Dysregulation Count represents the number of different tissues and/or immune cell types in which a given gene was found to be significantly dysregulated.

Shared expression trends were observed across immune cell types. We then applied a filter, retaining only genes that were significantly dysregulated in at least half of the cell types and consistently in the same direction. This approach identified a set of 118 genes that were consistently dysregulated across cellular environments. Further prioritization was based on the availability of high-resolution 3D crystal structures (≤2.6 Å) to enable accurate molecular docking and pharmacophore modeling, as well as the availability of sufficiently large datasets for virtual screening in LigandScout.

Gene	Regulation	GO Counts	KEGG	Dysregulation Count	Sum E	Resolution	Existing Library
**HSPA1A**	**up**	**11**	**1**	**9**	**21**	**available**	**Yes**
RPS27A	down	8	4	9	21	-	
HLA-A	down	0	11	9	20	-	
HLA-B	down	0	11	9	20	-	
HLA-C	down	0	11	9	20	-	
HLA-F	down	0	11	9	20	-	
RPL35	down	9	2	9	20	-	
RPS7	down	9	2	9	20	-	
RPL11	down	9	2	8	19	-	
RPL6	down	8	2	9	19	-	
RPS14	down	9	2	8	19	-	
RPS24	down	8	2	9	19	-	
**HSP90AA1**	**up**	**9**	**2**	**7**	**18**	**available**	**Yes**
RPL19	down	8	2	8	18	-	
RPL26	down	8	2	8	18	-	
RPL28	down	7	2	9	18	-	
RPS15A	down	7	2	9	18	-	
RPS19	down	9	2	7	18	-	
**TTN**	**up**	**9**	**0**	**9**	**18**	**available**	**Yes**
**HSPE1**	**up**	**8**	**0**	**9**	**17**	**not available**	**Yes**
RPL15	down	7	2	8	17	-	
EEF1D	down	7	0	9	16	-	
HLA-DPB1	down	0	8	8	16	-	
PRKDC	down	8	0	8	16	-	
**UBE2S**	**up**	**9**	**0**	**7**	**16**	**available**	**Yes**
**CHD2**	**up**	**6**	**0**	**9**	**15**	**available**	**Yes**
**CYCS**	**up**	**0**	**7**	**8**	**15**	**available**	**Yes**
**DNAJA1**	**up**	**8**	**0**	**7**	**15**	**not available**	**Yes**
**PLCG2**	**up**	**1**	**5**	**9**	**15**	**available**	**Yes**
TMA7	down	7	0	8	15	-	
**TRPM7**	**up**	**6**	**0**	**8**	**14**	**not available**	**Yes**
**ZNF207**	**up**	**4**	**0**	**9**	**13**	**unsure**	
**ATF4**	**up**	**2**	**1**	**9**	**12**	**unsure**	
B2M	down	0	4	8	12	-	
PYCARD	down	1	2	9	12	-	
RAC2	down	0	3	9	12	-	

ACTB	down	0	4	7	11	-	
ARPC2	down	0	2	9	11	-	
**DYNLL1**	**up**	**1**	**1**	**9**	**11**	**unsure**	
**RPL17**	**up**	**0**	**2**	**9**	**11**	**unsure**	
**TUBB4B**	**up**	**4**	**2**	**5**	**11**	**unsure**	
**ANXA2**	**up**	**0**	**1**	**9**	**10**	**unsure**	
ARPC3	down	0	2	8	10	-	
ATP5F1E	down	1	0	9	10	-	
CYBA	down	0	1	9	10	-	
GNAI2	down	0	2	8	10	-	
GTF3A	down	1	0	9	10	-	
MYL12A	down	0	2	8	10	-	
OXR1	down	1	0	9	10	-	
**RPS10**	**up**	**0**	**2**	**8**	**10**	**unsure**	
RPS2	down	0	2	8	10	-	
ABI3	down	0	0	9	9	-	
**ASH1L**	**up**	**0**	**0**	**9**	**9**	**unsure**	
CAP1	down	0	0	9	9	-	
CD37	down	0	0	9	9	-	
CD52	down	0	0	9	9	-	
CDKN2D	down	0	0	9	9	-	
**CEBPB**	**up**	**0**	**0**	**9**	**9**	**unsure**	
CIB1	down	0	0	9	9	-	
**CREM**	**up**	**0**	**0**	**9**	**9**	**unsure**	
**CYLD**	**up**	**0**	**0**	**9**	**9**	**unsure**	
DHRS7	down	0	0	9	9	-	
**DLEU2**	**up**	**0**	**0**	**9**	**9**	**unsure**	
**FAM107B**	**up**	**0**	**0**	**9**	**9**	**unsure**	
FXYD5	down	0	0	9	9	-	
GMFG	down	0	0	9	9	-	
**HIST1H4C**	**up**	**0**	**0**	**9**	**9**	**unsure**	
ICAM3	down	0	0	9	9		
IFITM2	down	0	0	9	9	-	
IFITM3	down	0	0	9	9	-	
IRF1	down	0	0	9	9	-	
LIMD2	down	0	0	9	9	-	
LSP1	down	0	0	9	9	-	
MAGOH	up	0	0	9	9	unsure	
MANF	up	0	0	9	9	unsure	
MED13	up	0	0	9	9	unsure	
MTRNR2L8	up	0	0	9	9	unsure	
NACA	down	0	0	9	9	-	
NEAT1	up	0	0	9	9	unsure	
NUFIP2	up	0	0	9	9	unsure	
OAZ1	down	1	0	8	9	-	
PCF11	up	0	0	9	9	unsure	
PFDN5	down	0	0	9	9	-	
PFN1	down	0	2	7	9	-	
PPP4R3A	up	0	0	9	9	unsure	
PSMB8	down	0	0	9	9	-	
PSME1	down	0	1	8	9	-	
RAB8B	up	0	0	9	9	unsure	
RPS26	up	0	2	7	9	unsure	
SKIL	up	0	0	9	9	unsure	

SNHG8	up	0	0	9	9	unsure	
SRSF2	up	0	0	9	9	unsure	
TMSB10	down	0	0	9	9	-	
TRA2B	up	0	0	9	9	unsure	
TXNIP	down	0	0	9	9	-	
VAMP8	down	0	0	9	9	-	
WAC	up	0	0	9	9	unsure	
YPEL5	up	0	0	9	9	unsure	
CFL1	down	0	1	7	8	-	
CORO1A	down	0	1	7	8	-	
GLS	up	0	0	8	8	unsure	
MAP1LC3B	up	0	2	6	8	unsure	
MBNL1	up	0	0	8	8	unsure	
NPIPB5	up	0	0	8	8	unsure	
POLR2J3-1	up	0	0	8	8	unsure	
PSMB9	down	0	0	8	8	-	
RGS19	down	0	0	8	8	-	
S1PR4	down	0	0	8	8	-	
SH3BGRL3	down	0	0	8	8	-	
ZBTB20	down	0	0	8	8	-	
ARHGDIB	down	0	0	7	7	-	
H2AFZ	up	0	0	7	7	unsure	
MTRNR2L12	up	0	0	7	7	unsure	
SLC3A2	up	0	0	7	7	unsure	
TLE4	up	0	0	7	7	unsure	
ABHD17A	down	0	0	6	6	-	
TMSB4X	down	1	0	5	6	-	
YPEL3	down	1	0	5	6	-	
